# Flexible 3D-Printed EEG Electrodes

**DOI:** 10.3390/s19071650

**Published:** 2019-04-06

**Authors:** Andrei Velcescu, Alexander Lindley, Ciro Cursio, Sammy Krachunov, Christopher Beach, Christopher A. Brown, Anthony K. P. Jones, Alexander J. Casson

**Affiliations:** 1 School of Electrical and Electronic Engineering, The University of Manchester, Manchester M13 9PL, UK; andrei.velcescu@postgrad.manchester.ac.uk (A.V.); alexander.lindley@student.manchester.ac.uk (A.L.); cirocursio@live.it (C.C.); christopher.beach@postgrad.manchester.ac.uk (C.B.); 2 Centre for Doctoral Training in Sensor Technologies and Application, Department of Chemical Engineering and Biotechnology, University of Cambridge, Cambridge CB2 1TN, UK; sm2205@cam.ac.uk; 3 Psychological Sciences, Institute of Population Health Sciences, University of Liverpool, Liverpool L69 3BX, UK; christopher.brown@liverpool.ac.uk; 4 Human Pain Research group, Division of Neuroscience and Cognitive Psychology, University of Manchester, Salford Royal NHS Foundation Trust, Manchester M13 9PL, UK; anthony.jones@manchester.ac.uk

**Keywords:** EEG, electrode, 3D printing

## Abstract

For electroencephalography (EEG) in haired regions of the head, finger-based electrodes have been proposed in order to part the hair and make a direct contact with the scalp. Previous work has demonstrated 3D-printed fingered electrodes to allow personalisation and different configurations of electrodes to be used for different people or for different parts of the head. This paper presents flexible 3D-printed EEG electrodes for the first time. A flexible 3D printing element is now used, with three different base mechanical structures giving differently-shaped electrodes. To obtain improved sensing performance, the silver coatings used previously have been replaced with a silver/silver-chloride coating. This results in reduced electrode contact impedance and reduced contact noise. Detailed electro-mechanical testing is presented to demonstrate the performance of the operation of the new electrodes, particularly with regards to changes in conductivity under compression, together with on-person tests to demonstrate the recording of EEG signals.

## 1. Introduction

**Figure 1:**
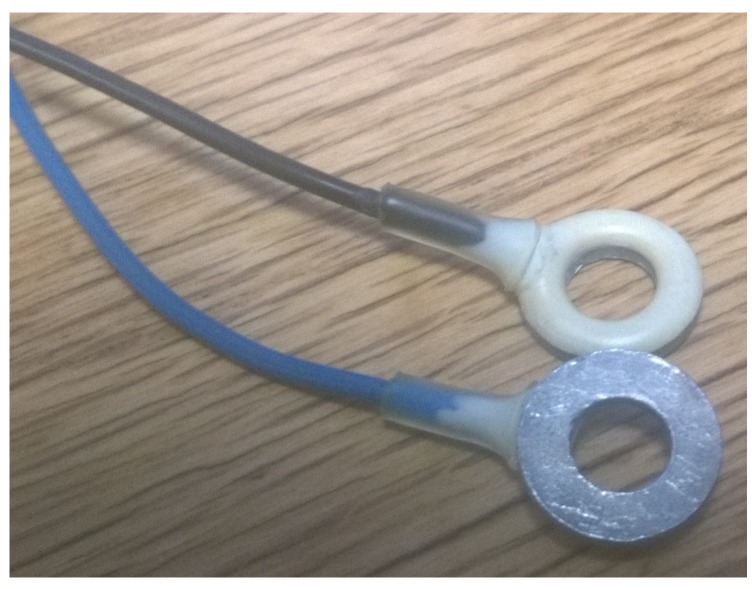
A conventional 1-cm disc EEG electrode made of sintered silver/silver-chloride.

EEG is a widely-used tool for the non-invasive monitoring of electrical signals in the brain and is used in applications from epilepsy diagnosis to brain–computer interfaces [1]. To collect the signal, conventionally, small metal disc electrodes such as those in Figure 1 are connected to the scalp and are held in place by either an electrode cap or by using an adhesive. A wide range of electrode shapes are possible, with 1 cm-diameter discs as in Figure 1 being the most common. A wide range of electrode materials [2] are also possible, with sintered silver/silver-chloride (Ag/AgCl) being the most widely used due to its biocompatibility, non-polarising nature and low contact noise and baseline drift [3].

While electrodes such as those in Figure 1 are very widely used, they still have a significant number of issues. In particular, they take a very long time to set up, and being flat, it is very difficult for the electrodes to make contact with the scalp rather than with any hair that might be present. A conductive gel is typically added to these electrode connections in order to help make a conductive bridge between the scalp and the bulk metal of the electrode. Although very important for getting the best signal quality, this gel takes a long time to apply, leaves a mess, dries out over time and is highly unpopular with both users and researchers.

**Figure 2:**
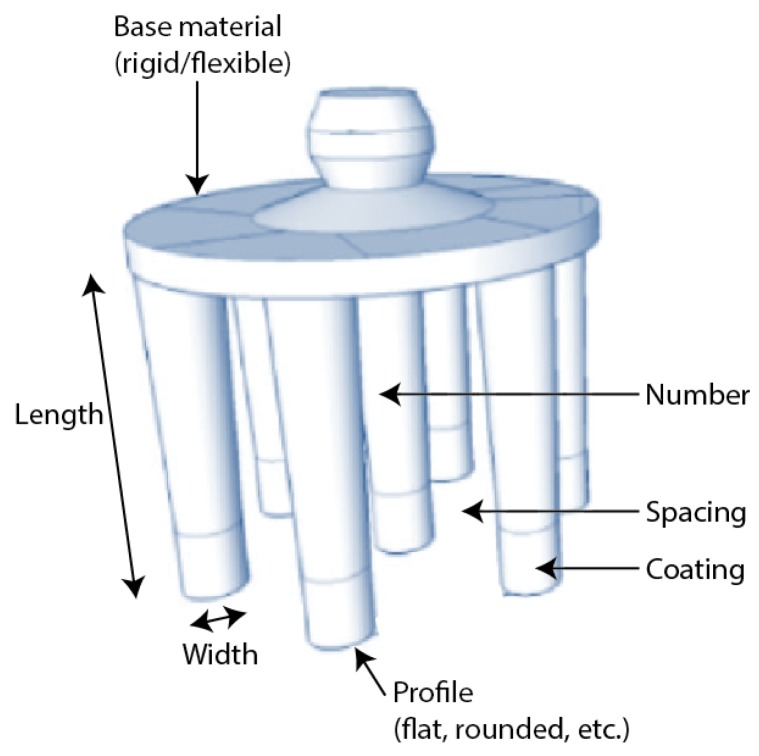
Personalisation parameters in fingered EEG electrodes for making a better connection to the scalp.

In recent years, *dry fingered* electrodes have emerged to help overcome these issues. Rather than being a disc that is likely to sit on top of any hair that is present, these electrodes have *fingers* or *prongs* to push apart the hair and make contact with the scalp. A number of such electrodes are commercially available, and a recent review was given in [4]. As shown in Figure 2, starting with a basic circular electrode, a wide number of different design parameters are available for fingered electrodes in order to give the best contact for each person, with different hair and skin types, and on different parts of the head.

There is thus significant potential for the personalisation of such electrodes, and the works in [5,6] have used 3D printing to allow personalised EEG electrodes to be fabricated in a near-real-time basis. The work in [5] used a high-performance (42-μm resolution) 3D printer to make a bed of 180 conical needles in an insulating acrylic-based photopolymer, with gold evaporated onto this base structure to make the electrodes conductive. The work in [6] used a desktop-grade 3D printer (0.5-mm resolution head used) to produce fingered 3D-printed electrodes similar in shape to that in Figure 2, which were then coated in silver. These electrodes were printed using a standard PLA plastic and so were rigid. This makes it possible for the fingers to *snap off* with use and can also be uncomfortable as the small finger tips press against the scalp.

This paper reports the design and performance of 3D-printed EEG electrodes, which are made using a flexible printing element to overcome the above issues, with a number of different shapes evaluated. The new electrodes have been coated in silver/silver-chloride to obtain better sensing performance compared to the earlier 3D-printed designs, which used silver to make the electrode conductive. They thus represent a second-generation 3D-printed electrode, building on our previous work reported in [6]. Section 2 describes the design and manufacturing of our new electrodes. Mechanical and electrical testing is then described in Section 3, with conclusions drawn in Section 4. As with our earlier designs, the base 3D printing files have been released under an open source license to allow others to re-use this work. Data availability details are given at the end of the paper.

## 2. Electrode Design and Manufacture

### 2.1. Electrode Base Fabrication

**Figure 3:**
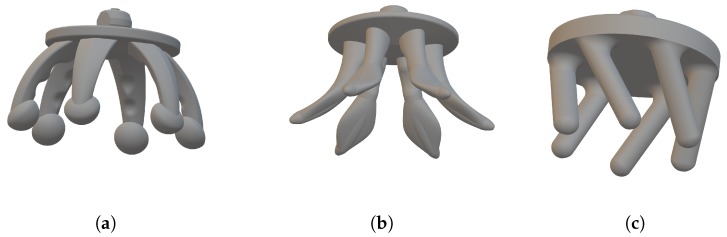
3D-printed electrode shapes investigated. All have a 1.5-mm snap connector on the upper side and are shown here with six fingers present. (**a**) Spider electrode. (**b**) Anti-spider electrode. (**c**) Spiny electrode.

A wide number of different electrode designs and configurations are possible via 3D printing, and in this work, we consider three *base* options as shown in Figure 3, all similar in starting shape to the Cognionics flexible electrode [7], g.tec g.SAHARA [8] and Neuroelectrics Drytrode [9] with a single ring of *fingers* around the outside. These base options are then manufactured with a range of finger numbers to get different levels of performance. All electrodes start with a flat base, which on one side has a 1.5-mm snap connector printed for connecting the electrode to standard electro-physiological recording equipment. The electrodes then differ in the shapes of the fingers that connect to the other side of the base.

Figure 3a shows the *spider* electrode type with six fingers. Here, the fingers are convex such that the finger tips spread outwards when they are pushed down, pushing hair out of the way. This is intended to be similar to the shape of the Cognionics electrode [7], which is available commercially. Our electrode design has a new ball shape at the end of each finger to increase the potential contact area available. The finger also has small parts taken out of it along the length, such that the cross-sectional area is not constant, in order to increase the mechanical flexibility after printing. Figure 3b shows the *anti-spider* electrode type where fingers are concave such that when pushed down, the inner side of the leg makes contact with the scalp. This inner surface has been flattened in our design compared to having a circular cross-section in order to increase the potential contact area available. Figure 3c shows the *spiny* electrode type where fingers are cylindrical, projecting out at an angle of 30 degrees from the base. When this electrode is pressed down, the fingers do not spread, but rather, the electrode collapses in on itself, so the electrodes act as a mechanical buffer.

These three base shapes were printed using a desktop-grade 3D printer, a Lulzbot Mini 3D printer with settings: height = 0.24 mm, temperature = 223, printing speed 12 and travel speed 200. The electrodes were printed with the prongs facing upwards, with an automatically-generated support structure added to the printing/filament profile as a 15% infill support structure. A commercially-available flexible TPU (thermoplastic Polyurethane) printing filament (NinjaFlex Semiflex) was used, and after printing, a lighter was lightly passed near to the electrodes as they were removed from the printer to remove residual stringing.

**Figure 4:**
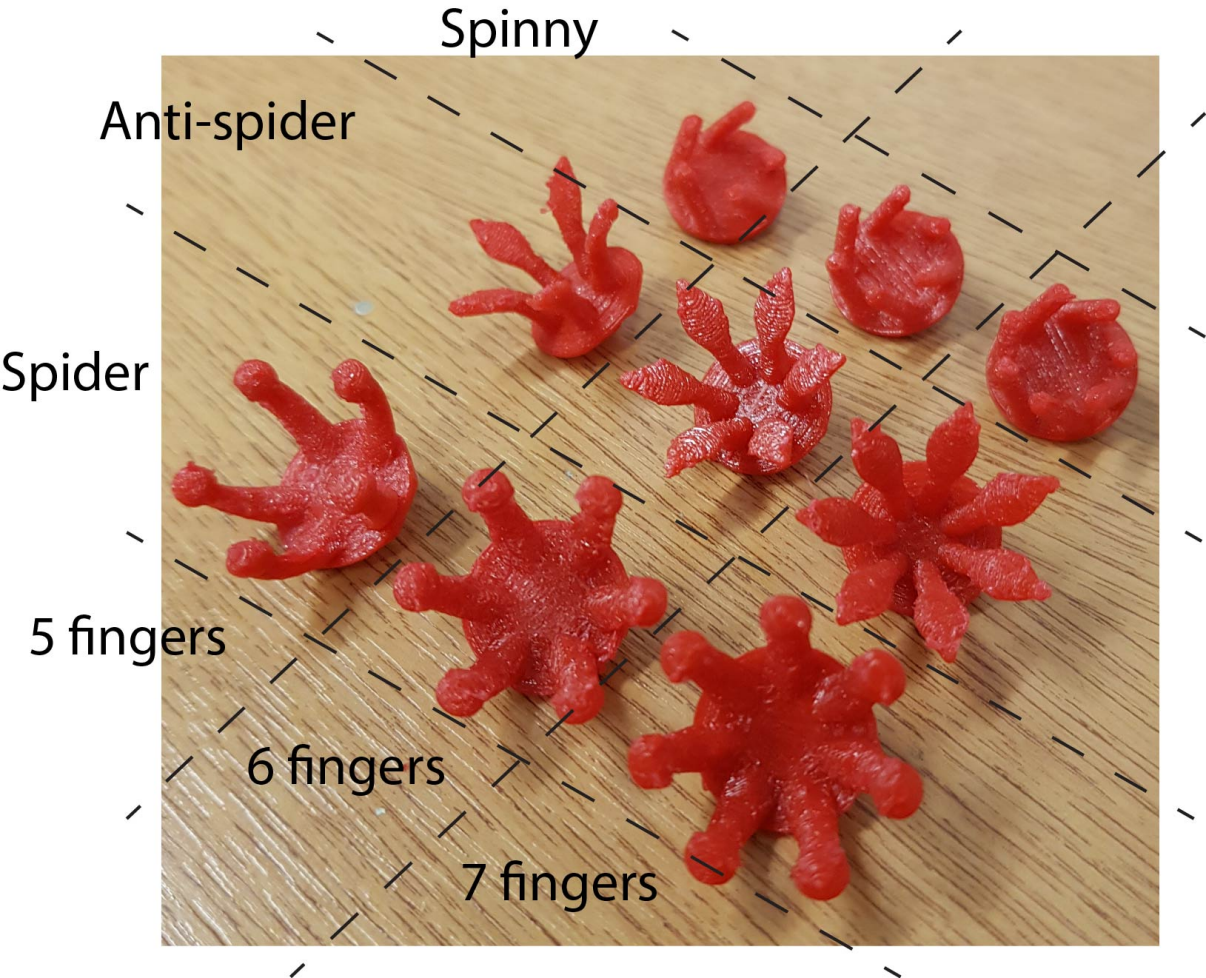
Nine different electrode configurations investigated here after 3D printing.

A picture showing the base printed electrodes is given in Figure 4. This shows the three electrode types, each printed with 5, 6 and 7 fingers, giving 9 electrode configurations in total to test. For each configuration, the finger shape was the same, the only difference being the angular spacing of the fingers on the base, which decreased uniformly as the number of fingers increased.

### 2.2. Electrode Coating

The base electrodes shown in Figure 4 are non-conductive and so are not directly suitable for measuring EEG and first need activating by depositing a conductive layer on top. Previously, we used silver ink for this [6], as it is readily available and inexpensive in small quantities and has previously been used for EEG electrodes in [3]. However, silver has poor long-term stability as a skin–electrode interface [3], with silver/silver-chloride giving less noise and better stability [3]. For improved sensing performance, we used silver/silver-chloride ink available from Creative Materials [10], matching the material used in conventional EEG electrodes. This silver/silver-chloride ink is medical grade and specifically designed for the collection of electro-physiological bio-signals. We coated the whole of the electrode, unlike approaches such as the Cognionics electrode [7] which only has the tip coated, as our desktop-grade, flexible, 3D printer element was not conductive in its own right. This comes at the cost of needing much more Ag/AgCl coating than approaches that only cover the electrode tip.

**Figure 5:**
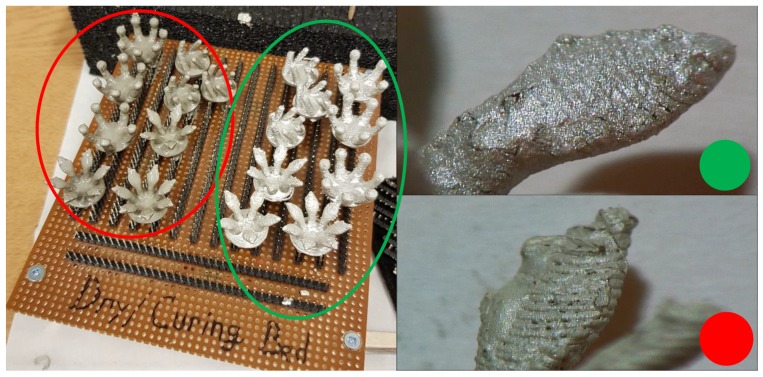
Coated electrodes, with a ×40 zoom. Green: Ag/AgCl used for the results in Section 3. Red: silicone Ag/AgCl, which gave a poor adhesion to the 3D-printed base.

Figure 5 shows two sets of electrodes that have been coated in two different inks: Ag/AgCl (mixture 113-09 from Creative Materials) and Silicone Ag/AgCl (mixture 126-49 from Creative Materials for highly-flexible substrates). These were applied in a 50/50 mix of paint and thinner, using dip coating to ensure that the electrode was fully covered. The electrodes were then placed on the curing bed seen in Figure 5 for 12 h after the ink application. Fifteen minutes of curing was then performed. For silicone Ag/AgCl, the curing was done at 160 degrees Celsius, keeping the temperature below the 168 degrees Celsius melting point of the NinjaFlex Semiflex filament, and the Ag/AgCl curing was done at 100 degrees Celsius. The silicone Ag/AgCl was found to have a poor adhesion to the 3D-printed filament and would require many hours to dry fully even after the curing process, leaving a poor quality surface on the finished electrode. Based on this, results in Section 3 only consider the use of the Ag/AgCl ink.

### 2.3. Electrode Summary and Test Methods

The final electrodes used for testing are summarised in Table 1. This includes estimates of the total contact surface area available from each design when they are fully pressed down, which affects the performance of the electrode [11]. For comparison to other electrodes, we also include measured results from a commercially-available passive disc Ag/AgCl electrode from EasyCap (Herrsching, Germany) and the *Foretrode* dry EEG electrode for use on the forehead from Neuroelectrics (Barcelona, Spain).

**Figure 6:**
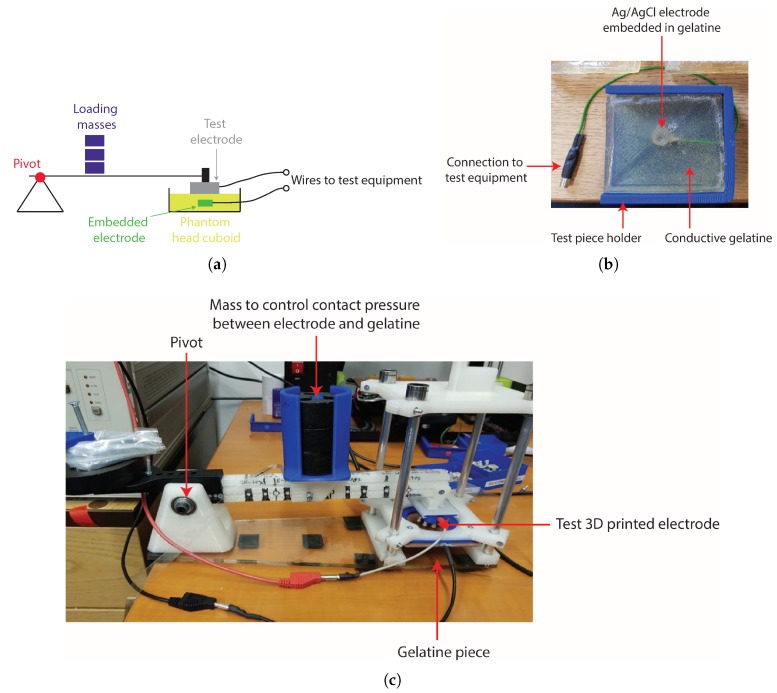
Test setup used. (**a**) Pivot structure used to control the force/pressure of the contact between the electrode and the gelatine test piece. (**b**) Conductive gelatine used as a phantom head, here set in the shape of a cuboid with an embedded reference electrode. (**c**) Photograph of the arrangement.

In order to measure the electrical and mechanical properties of EEG electrodes in a controlled way, the work in [3] introduced the use of a conductive agar as a phantom head model, which replicates the ionic conductors present in the head and scalp. This was extended in [12] to use ballistic-grade gelatine, and we made use of this approach with the tests in Section 3, using a phantom head model previously reported in [6,11,13]. A 30% gelatine to 70% water mixture was used, and unlike [11,13], where the gelatine was set in the shape of a physical head, in this study, the gelatine was set as 6 × 6 cm cuboids, allowing them to be placed into a mechanical test structure; see Figure 6. A conventional silver/silver-chloride electrode was set into the gelatine as shown in Figure 6b to act as a reference electrode, with the test 3D-printed electrode placed on the surface of the gelatine cuboid. This could then be placed in a mechanical test set, with one configuration shown in Figure 6c, where weights could be added to change the pressure/loading with which the test electrode was pressed against the gelatine piece. The mass used to load the test electrode was varied between 150 g and 400 g, with the resulting pressure exerted on the electrode–gelatine (electrode–skin) interface, which will depend on the contact area of the electrode, given in Table 1. This range of masses, and resulting pressures, was selected to match the range of comfortable pressures for EEG electrodes in on-person tests reported in [14] with our estimated contact areas in Table 1.

**Figure 7:**
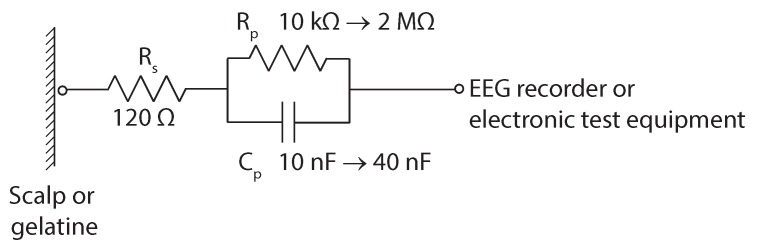
Wet silver/silver-chloride electrode model from [15]. $R_{s}$ is a series resistor modelling the bulk electrode impedance and the series component of the contact impedance, while $R_{p}$ and $C_{p}$ model the rest of the contact impedance.

Contact impedance of the different electrodes was measured using an Agilent 4284A Impedance Analyzer, between 20 and 1000 Hz, with the 3D-printed electrode as one terminal and the electrode inside the gelatine model as the other terminal. A 135-g mass was used for all tests. These contact impedance magnitude and phase results were compared against the electrical model of a wet silver/silver-chloride electrode from [15] and shown in Figure 7. This is made up of a series resistor ($R_{s}$) with a value of 120 Ω, a parallel resistor ($R_{p}$) with a value between 10 kΩ and 2 MΩ and a parallel capacitor ($C_{p}$) with a value between 10 nF and 40 nF [15].

If the base electrode material is flexible to increase comfort, it is essential that the conductivity of the electrode material remain approximately constant under different amounts of compression/tension [16]. Without this, slightly different signals will be collected depending on how the EEG is put on; if the cap slightly tighter or slightly looser, changing the amount of compression present. To study this, three different tests of mechanical performance are reported here. Firstly, pictures are given of the physical deformation of the electrodes when pressed against a fixed surface to show how the legs spread. Secondly, the DC bulk impedance (resistance) of the electrodes is shown as they are loaded at 50 g, then 350 g, and then 50 g again to show any hysteresis effects. Finally, a test of the contact impedance magnitude and phase at 35 Hz is performed, matching the frequency used by the SIGGI II EEG impedance meter (Easycap, Germany) for on-person contact impedance measurements, as the loading mass is swept from 0–400 g. For brevity here, this last result is reported only for the spider electrode with seven fingers. In all cases where the mass was varied, the electrode was allowed to settle for five minutes before a reading was taken to remove any transient effects.

The contact noise of each electrode was measured by performing a two-electrode EEG measurement with a camNtech actiwave EEG recorder (camNtech, Cambridge, UK), with the 3D-printed electrode and silver/silver-chloride electrode inside the gelatine acting as the two contact points. This records the residual electrical noise when no EEG signal is present. The recorded signal is thus only the electrode contact noise and the instrumentation noise that will be common to all tests. A reference recording using a conventional EEG electrode was included to quantify this common noise. In all cases, 10-bit, 1024-Hz sampling was used, with three-minute recordings band-limited to 100 Hz. The average RMS of the recorded signals in the 0.3–100 Hz range, with a 150-g loading mass, and popcorn noise greater than 15 μV removed, is then reported. As short-term recordings were used, we did not extract the drift rate of the electrodes from this noise test and did not consider how the drift rate varies for the different types of electrodes under different amounts of pressure. This should be considered as a limitation of the current work.

Finally, to demonstrate EEG recordings, a functional test where the electrodes were used on a person is presented. Pairs of electrodes were set up in turn, with one seven-finger electrode over FCz and one six-finger electrode of the same type over Oz, with the electrodes held down using a standard EEG cap. A two-electrode EEG recording using the camNtech actiwave EEG recorder (camNtech, Cambridge, UK) was then performed. All signals were sampled at 1024 Hz, 10-bit resolution and band-limited from 1–30 Hz for presentation. To give recognisable signals in the time domain traces, the participant sat stationary and was asked to shut his/her eyes after 30 s, allowing spontaneous alpha activity to be observed at the back of the head. All procedures performed involving human participants were in accordance with the ethical standards of the institutional and/or national research committee and the 1964 Helsinki declaration and its later amendments or comparable ethical standards. Informed consent was obtained from all individual participants included in the study. The research was approved by the University of Manchester research ethics committee, Number 2018-4015-5913.

## 3. Results and Discussion

### 3.1. Contact Impedance

**Figure 8:**
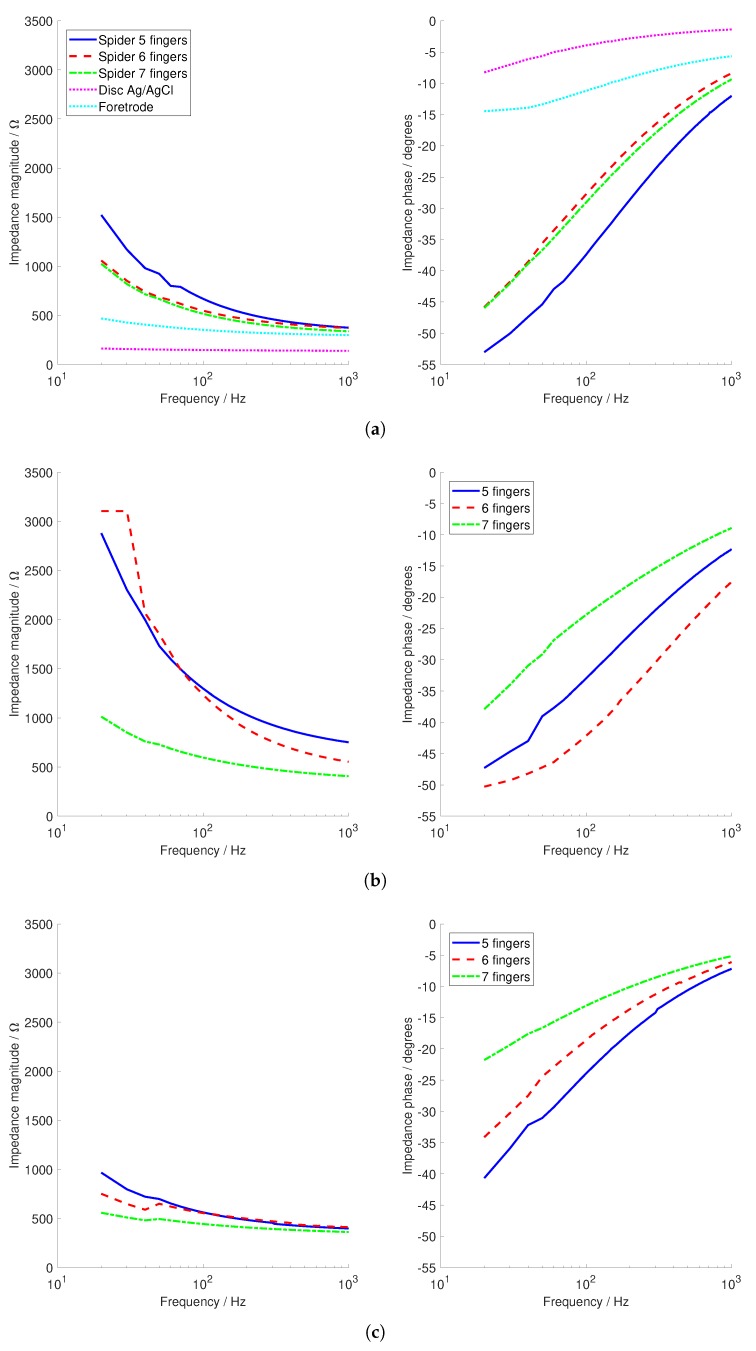
Contact impedance (magnitude and phase) for the different electrode configurations. (**a**) Spider electrode and the Ag/AgCl disc and Foretrode comparison electrodes. (**b**) Anti-spider electrode. (**c**) Spiny electrode.

The contact impedances to the phantom head for a 135-g loading are shown in Figure 8. Connections to the phantom head did not have hair and similar application obstacles, and so, the contact impedances were generally low; lower than those with our previous dry 3D-printed electrodes, which were in the range of 5–10 kΩ [6] and below the 10 kΩ limit typically used for passive electrode EEG recordings, but higher than the Ag/AgCl disc and Foretrode comparison electrodes. All of the electrodes had a similar pattern with the contact impedance reducing at higher frequencies, with more fingers generally giving lower contact impedance. The spiny electrode had the lowest contact impedance, although it was similar in magnitude to the spider electrode. The anti-spider electrode had approximately twice the contact impedance.

The phase of the contact impedance is also shown in Figure 8, with more fingers resulting in less phase change. The value of the phase change increased in magnitude at low frequencies, with up to −50 degrees present. In contrast, the measured phase change of the reference commercial electrodes was smaller, only up to −15 degrees. Nevertheless, these phase changes were in-line with those expected from standard Ag/AgCl electrodes, with phase changes of up to −80 degrees given by the electrode model from Figure 7 [15]. The phase change was approximately linear, and the resulting mean group delays are given in Table 2. The values for the 3D-printed electrodes were approximately double those obtained for the electrode model from [15], where the largest group delay was 59 μs (with $R_{p}=2\;M\Omega$ and $C_{p}=10\;\mathrm{nF}$) and four-times the measured values for the disc Ag/AgCl and Foretrode electrodes. This increased group delay will lead to greater timing distortion of the EEG waveform, but the effect was sub-100 μs for most cases, so it was not substantial to compare to EEG evoked responses, which typically have durations of hundreds of milliseconds.

### 3.2. Mechanical Performance

**Figure 9:**
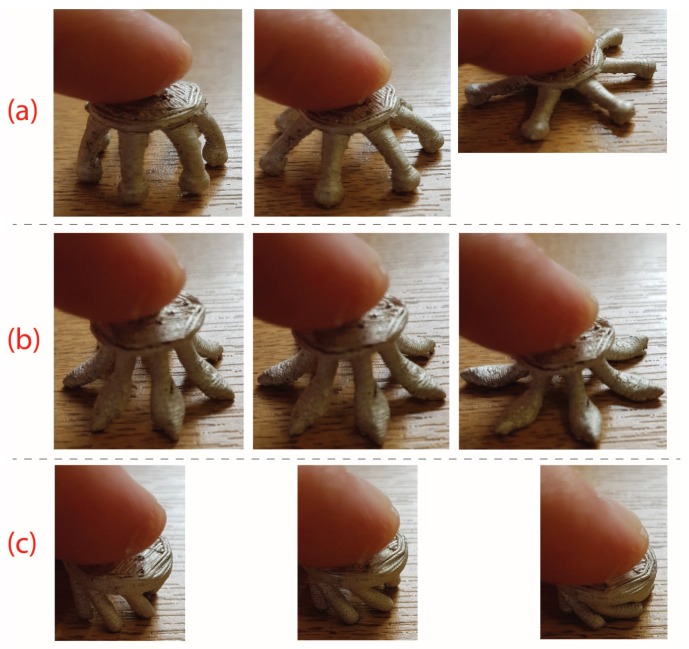
Physical deformations of the different electrodes when pressed against a fixed surface show the different shapes in which the fingers spread. (**a**) Spider electrode. (**b**) Anti-spider electrode. (**c**) Spiny electrode.

The physical deformations of the different electrodes when pressed against a fixed surface are shown in Figure 9. The spider and anti-spider electrodes spread outwards, while the spiny electrode collapsed in on itself. The bulk resistances of the commercial disc Ag/AgCl electrode was 1.1 Ω including the long connection wire and for the Foretrode, 0.3 Ω. In comparison, the bulk resistances of the flexible electrodes are given in Table 3, where the mass pressing the electrode down was varied cyclically between between 50 g and 350 g.

In all cases, the resistance was low, below 2 Ω, showing that sufficient conductivity was provided by the Ag/AgCl coating for this resistance to be insignificant compared to the typical kΩ contact impedance obtained from the scalp connection. The resistance present depends on the loading used, with hysteresis present such that the resistance after heavy loading is not exactly the same as that before heavy loading. However, this effect was small and below 1 Ω in all cases. This hysteresis was equivalent in magnitude to the conductive polymer electrodes presented in [16], which were specifically designed to have a consistent level of conductivity at different load levels (Figure 4 in [16] showed a 10 Hz contact impedance measurement, with approximately 1 Ω hysteresis present for a conductive polymer with 7% filler used).

**Figure 10:**
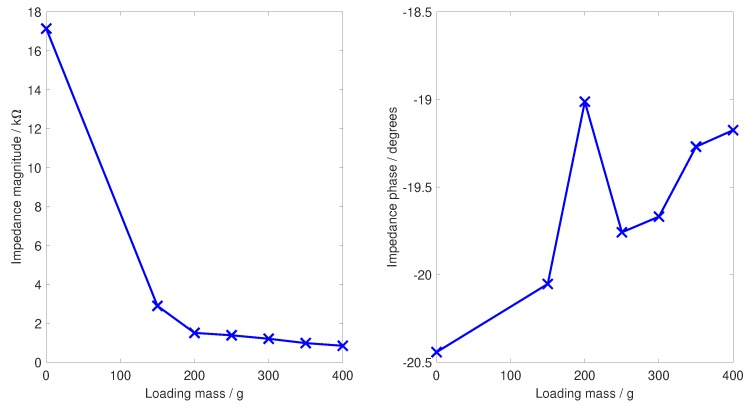
Contact impedance for the spider seven-finger electrode, measured at 35 Hz to match on-person contact impedance readings as the contact loading is varied across the comfortable range.

A similar trend is seen in Figure 10, which shows the contact impedance at 35 Hz, illustrated here only for the spider seven-finger electrode, as the loading mass was swept from 0–400 g. With no force pressing against the electrode, the contact impedance was high; some level of pressing against the head phantom scalp was needed to obtain a connection. Within the range of loadings from 150–400 g, corresponding to the comfortable pressure range from [14], the contact impedance fell with increased pressure and was always below 3 kΩ. The phase change for any level of loading was less than two degrees from the no loading case, with the peak at 200 g attributed to measurement error at the limit of our impedance analyser unit.

To date, we have not been able to perform long-term cyclic loading tests, where the above analysis is repeated over hundreds or thousands of cycles, and this should be taken as a limitation of the current study. After prolonged use, the Ag/AgCl coating can begin to crack, particularly at the base of the electrodes where the fingers connect and flex. This presents no limitation for short-term use, but will be the limiting factor for the same electrodes being re-used many times. Note also that there is no clear trend in bulk impedance in Table 3 and the number of fingers present. We attribute this to intrinsic variances in the manufacturing process between different electrodes, for example the Ag/AgCl coating quality and actual manufactured size of each finger, both of which will vary slightly. To date, these variances have been small (as seen in Table 3) and do not affect usability, but future work should investigate the manufacturing repeatability and the change in performance between different manufacturing and coating runs.

### 3.3. Noise Performance

The noise performance of the electrodes between 0.3 Hz and 100 Hz is summarised in Table 4. The worst case noise was for the spider electrode with six fingers, where 3.3 μVrms of noise was present. In comparison, the measured noise of a standard EEG electrode using the same test setup was 0.9 μVrms and for the Foretrode, 0.9 μVrms. The silver/silver-chloride 3D-printed electrodes thus had more noise than a traditional bulk metal silver/silver-chloride electrode, but less than our previously-reported silver 3D-printed electrodes where the noise was 5–15 μVrms, depending on the electrode configuration used. From the spectrogram of the recorded noise, flicker noise dominated below approximately 10 Hz, with white noise present above this frequency.

### 3.4. EEG Recordings

**Figure 11:**
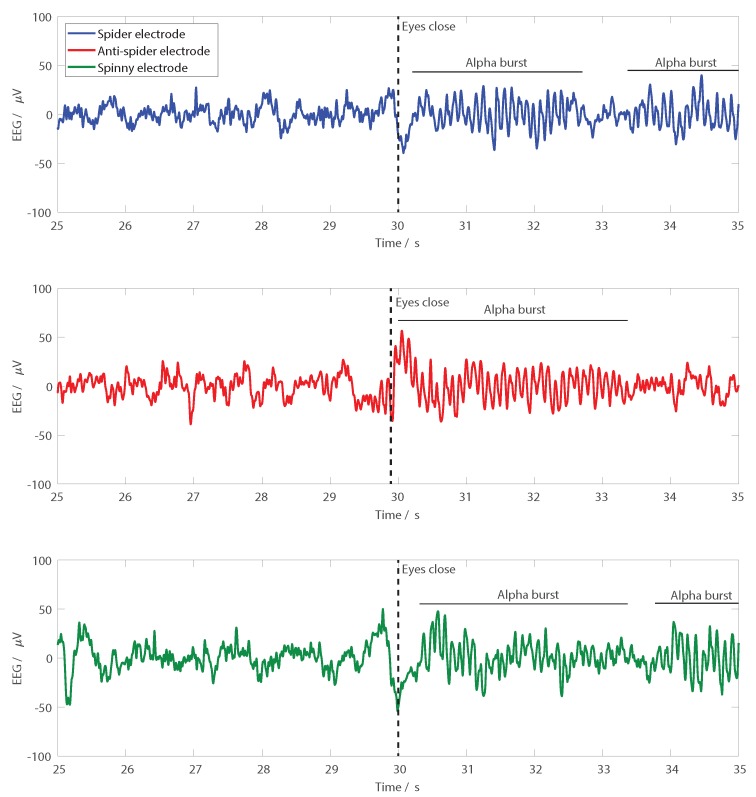
Example EEG recordings from Oz with the three different electrode types. Participants were asked to close their eyes at the 30-s mark, and clear bursts of alpha activity are seen following the eyes closing with all of the electrode types.

Figure 11 shows example EEG recordings performed using the different electrode types. In all cases, the participant was asked to close their eyes at the 30-s mark, and spontaneous alpha activity was then seen at the back of the head. Note that these recordings were performed at different points in time. To avoid the distortion effects from reference electrodes discussed in [11], in each electrode application, only one type of electrode was used. As such, the three traces in Figure 11 were not expected to be identical. They nevertheless demonstrated that EEG recordings can be performed with all three different types of electrode.

## 4. Conclusions

This paper has presented nine different configurations of flexible 3D-printed EEG electrodes. These represent a second generation 3D-printed electrode over our previous work, with the electrodes now being flexible (rather than rigid) to improve comfort, and having improved EEG sensing performance via the use of a silver/silver-chloride coating. The new electrodes have reduced contact impedance and reduced contact noise compared to our previous 3D-printed electrodes, with both factors investigated using a phantom head model, which allowed the contact impedance changes at different contact pressures to be characterised. The potential for custom manufacturing of electrodes opens many new opportunities for personalisation, as well as using different electrodes for different parts of the head and for different people. However, at present, we do not have a defined set of rules for how we would select a differently-shaped electrode for different people or for different parts of the head. Future work will focus on the comfort testing of different electrode shapes and structures, to optimise this personalisation, as an important design factor for eventual electrode selection in addition to the electro-mechanical performances reported here.

## Figures and Tables

**Table 1:** Summary of the 9 electrode configurations used in this work.

Electrode Type	Fingers	Approximate Contact Surface Area (mm^2^)	Pressure with 150 g Loading (kPa)	Pressure with 400 g Loading (kPa)
Spider	5	106.3	1.39	03.69
	6	127.5	1.15	03.08
	7	148.8	0.99	02.64
Anti-spider	5	101.4	1.45	03.87
	6	121.7	1.21	03.23
	7	142.0	1.04	02.76
Spiny	5	032.1	4.59	12.24
	6	038.5	3.83	10.20
	7	044.9	3.28	08.75

**Table 2:** Mean group delay for the different electrodes.

Electrode Type	Fingers	Group Delay (μs)
Spider	5	116
	6	106
	7	104
Anti-spider	5	091
	6	093
	7	082
Spiny	5	095
	6	079
	7	047
Disk Ag/AgCl	–	020
Foretrode	–	025

**Table 3:** Bulk resistance of electrodes when loading is varied from 50–350 g and back to 50 g.

Electrode Type	Fingers	Resistance with Loading Mass *m* (Ω)		
		m=50 g	m=350 g	m=50 g
Spider	5	1.89	0.29	1.11
	6	0.86	0.28	0.91
	7	1.14	0.40	0.83
Anti-spider	5	0.20	0.12	0.21
	6	0.56	0.18	0.66
	7	0.31	0.18	0.32
Spiny	5	0.31	0.13	0.26
	6	0.21	0.17	0.24
	7	1.38	0.37	0.99

**Table 4:** Noise performance of the electrodes with a 0.3–100-Hz bandwidth.

Electrode Type	Fingers	Noise (μVrms)
Spider	5	2.4
	6	3.3
	7	1.4
Anti-spider	5	3.1
	6	2.4
	7	3.0
Spiny	5	2.2
	6	1.9
	7	1.9
Disk Ag/AgCl	–	0.9
Foretrode	–	0.9
